# Confined placental mosaicism of Duchenne muscular dystrophy: a case report

**DOI:** 10.1186/s13039-020-00520-3

**Published:** 2020-12-17

**Authors:** Max Winerdal, Eini Westenius, Michaela Granfors, Maria Pettersson, Erik Iwarsson

**Affiliations:** 1 Department of Clinical Genetics, L4:03, Karolinska University Laboratory, Karolinska University Hospital, 171 76 Stockholm, Sweden; 2 Division of Obstetrics, Department of Women’s Health, Karolinska University Hospital, Stockholm, Sweden; 3 Clinical Epidemiology Division, Department of Medicine, Solna, Karolinska Institutet, Stockholm, Sweden; 4 Department of Molecular Medicine and Surgery, Karolinska Institutet, Stockholm, Sweden

**Keywords:** Mosaicism, Placenta, Chorionic villus sampling, Amniocentesis, Chromosome fragile site, Duchenne muscular dystrophy, DNA copy number variation, X chromosome, Prenatal diagnosis, Confined placental mosaicism

## Abstract

**Background:**

Small copy number variations confined to the placenta are extremely rare findings in chorionic villus sampling, nonetheless of great clinical importance. To the best of our knowledge, this is the first reported case of confined placental mosaicism for an intragenic Duchenne muscular dystrophy (*DMD*) gene deletion.

**Case presentation:**

We describe a pregnant woman where confined placental mosaicism for an intragenic *DMD* deletion was detected. She was referred for a chorionic villus sampling due to an increased risk of trisomy 21 derived from combined first trimester screening. Rapid aneuploidy detection showed a male fetus with normal results for chromosomes 13, 18 and 21. A chromosomal microarray demonstrated a deletion of exons 61–62 in the *DMD* gene in approximately 50% of the cells. A follow-up analysis on amniotic cells showed a normal result for the *DMD* gene. Hence, confined placental mosaicism was confirmed.

**Conclusions:**

We propose tissue specific fragile sites as a possible theoretical mechanism for the formation of submicroscopic copy number variations and highlight that the finding of *DMD* deletion mosaicism in a chorionic villus sample might be isolated to the placenta. Therefore, confirmation by amniocentesis is of crucial clinical importance to avoid misdiagnosis of the fetus.

## Background

Duchenne muscular dystrophy (DMD) with X-linked recessive inheritance is the most common myopathy in children and affects one in every 3500 boys. It is caused by mutations in the dystrophin gene located on the short arm of the X chromosome (Xp21). Two-thirds of DMD cases are caused by a large deletion of one or several exons in the *DMD* gene. Duplications, single nucleotide variants (SNVs), smaller deletions or insertions, and splice site changes account for the remaining cases. In one-third of the cases with DMD, the pathogenic variant occurs de novo.

Confined placental mosaicism (CPM) develops as a result of a postzygotic mutational event and is defined as the presence of a chromosome aberration in a mosaic form in the extra-embryonic tissue and absence in the fetal tissue. CPM is observed in approximately 2% of chorionic villi samples analyzed with conventional karyotyping [1–4]. Three studies evaluating CMA and the detection of mosaicism in prenatal samples estimated the prevalence of mosaicism to between 1.8 and 4.1%, suggesting higher detection rates with CMA than conventional karyotyping [5–7].

When a chorionic villus sampling (CVS) indicates mosaicism, it is recommended to perform an amniocentesis (AC) to exclude a true fetal chromosome aberration. Even in the case of a normal amniocentesis, genetic counselling can be challenging due to the possible pregnancy complications CPM can cause. Placental dysfunction leading to intrauterine growth restriction and other complications, such as pregnancy loss, pre-term births and newborns small for gestational age have been reported [8–11]. The magnitude of clinical symptoms caused by CPM for a submicroscopic CNV depend on the timing of the mutational event, which cell lineages are affected, the phenotypical consequences of the aberration and the impact on selection and cell viability [11, 12]. CPM can involve different types of numerical and/or structural chromosome aberrations, where autosomal trisomies are the most common abnormality [1, 2, 13]. There is a vast amount of data on CPM for aneuploidy and structural chromosome aberrations [3, 14], but very few cases of CPM for submicroscopic copy number variations (CNV) < 10 Mb [5, 7, 15, 16]. To our knowledge, the largest study regarding CMA detecting mosaicism for submicroscopic CNVs in prenatal samples is so far by Lund et al. who identified 93 cases of mosaicism out of 2288 (4.1%) prenatal CMAs on uncultured chorionic villi [7]. In their study, 18.3% of the mosaicism cases involved submicroscopic CNVs, they confirmed the possibility of true fetal mosaicism (TFM) of a submicroscopic CNV in three new cases and they found no statistically significant difference in the prevalence of CPM and TFM when comparing mosaicism involving CNVs and whole chromosomes. There are eight previous cases of CPM for a submicroscopic CNV on the X chromosome reported in the literature [5, 7, 15]. To the best of our knowledge, this is the first report of a case of CPM for a *DMD* gene deletion.

## Case presentation

Our patient, a 34-year-old pregnant woman, G3 P2, without any known medical or hereditary history went through a combined first trimester screening with a nuchal translucency of 2.5 mm and a risk assessment of 1:4 for trisomy 21. As per local routine, when the risk is ≥ 1:50, our patient was offered a chorionic villus sampling (CVS), which was performed at gestational age (GA) 13 + 1.

**Fig. 1:**
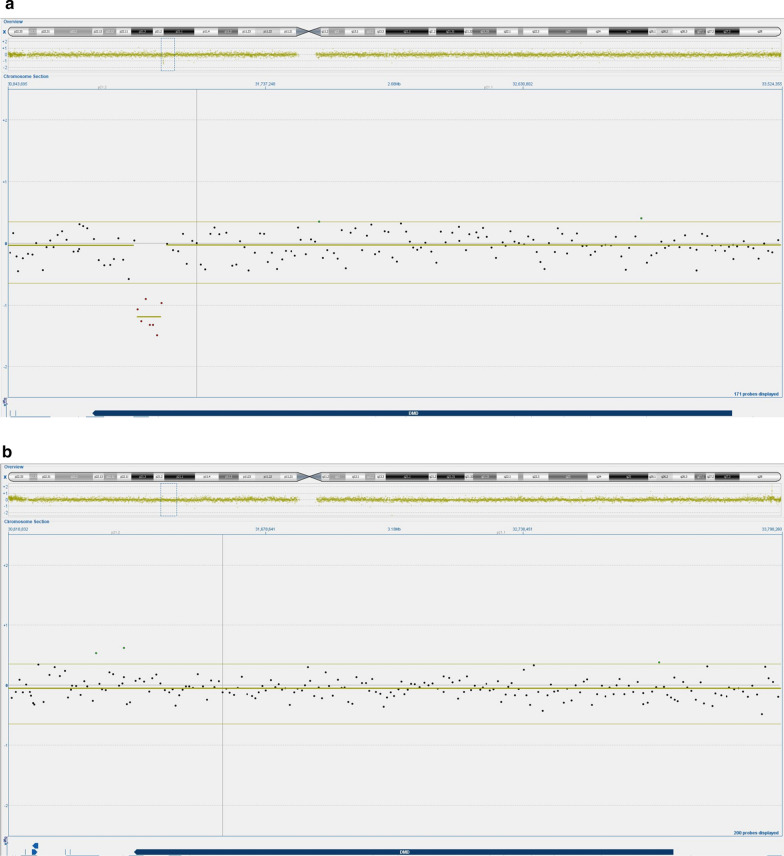
**a** Chromosomal microarray performed on chorionic villi showed a deletion of exons 61–62 in the *DMD* gene (Xp21.2, ≈ 84 kb) in approximately 50% of chorionic villi. **b** Chromosomal microarray performed on amniotic cells showed a normal result for chromosome X

Rapid aneuploidy detection using quantitative fluorescence polymerase chain reaction (QF-PCR) with a panel of PCR primers specific to chromosomes 13, 18, 21, X and Y showed a normal result and confirmed that the fetus was male. Chromosomal microarray (CMA) using array comparative genomic hybridization (aCGH) demonstrated a deletion of exons 61–62 in the *DMD* gene (Xp21.2, ≈ 84 kb) in approximately 50% of the cells (Fig. 1a). Multiplex ligation-dependent probe amplification (MLPA) confirmed the *DMD* deletion and substantiated the mosaic pattern.

Due to male gender of the fetus, X-linked recessive inheritance and the possibility of CPM, an amniocentesis (AC) was performed. CMA of DNA extracted from uncultured amniotic cells showed a normal result for chromosome X (Fig. 1b), suggesting that the microdeletion had arisen during placentation and was confined in mosaic state to the placenta. No further genetic analysis was performed. The second trimester ultrasound scan and an extra ultrasound at 35 weeks of gestation turned out normal. A healthy baby boy was born at GA 40 + 6, with a birth weight of 3535 g and normal routine checks and follow-up visits until the age of 4 months.

## Discussion and conclusion

Awareness of the fact that a *DMD* deletion mosaicism in a CVS might be isolated to the placenta is of great clinical importance. Such a finding requires confirmation by AC in order to avoid errors in the assessment of genetic status in the fetus. This will be even more important to acknowledge since some NIPT platforms performance are rapidly approaching that of CMA. This enables readily detection of submicroscopic CNVs, where false positive results due to CPM must be excluded. When mosaicism for a chromosome aberration has been identified in a CVS, a normal AC suggests CPM and a non-carrier status of the fetus. However, a low-level true fetal mosaicism (TFM) is difficult to fully exclude. Nonetheless, a study by Lund et al. [7] indicates that the risk for a symptomatic low-grade TFM should be very low when placental mosaicism is demonstrated and the AC turns out normal.

For the eight previously reported cases of CPM for a submicroscopic deletion on the X chromosome, there are three cases of *FMR1* deletion, two cases of *IL1RAPL1* deletion and three cases of *STS* deletion [5, 7, 15]. Notably, the *DMD* gene is located proximally alongside the *IL1RAPL1* gene within the fragile site FRAXC [17]. Furthermore, the *FMR1* gene is located in the fragile site FRAXA and the *STS* gene in FRAXB [18]. Thus, fragile sites prone to de novo mutations seem to emerge as hotspots for submicroscopic deletions confined to the placenta. Investigation of fragile sites has revealed increased susceptibility in preferentially large genes, genes with a late replication timing and a paucity of replication origins during replication stress [19, 20]. Intriguingly, the *DMD* deletion in our sample lies in a transcription factor binding site, in an intersection region for early and late replication timing as seen by Repli-seq data [21]. There is indirect indication of genomic instability in small (< 3 Mb) CNVs in other chromosomes than the X chromosome as well [6]. The same CNV regions found in CPM are repeatedly found as constitutional (mainly de novo) mutations or as acquired mutations in cancers. Some CNV regions previously observed in CPM have, when constitutionally expressed, been reported to be susceptible to nonallelic homologous recombination [22], suggested as a fragile site [23] or as mutational hotspots [24, 25]. In contrast to other CNV regions found in CPM, subtelomeric rearrangements in the 9q34.3 region display nonrecurrent breakpoints [25]. Nonetheless, high rearrangement rates are seen in specific parts of this region, suggesting a different fragility mechanism. Furthermore, gender specific differences of rearrangement types have been observed in this region, which speculatively could be related to imprinting status. Since active transcription is a prerequisite for tissue type specific fragile site formation, only genes actively expressed in the tissue would be expected to be at increased risk for a submicroscopic deletion in placental tissue. CNVs in CPM larger than approximately 3 Mb are more likely due to translocations or other chromosomal rearrangements, since other concomitant rearrangements or a sibling with chromosome aberration were observed to be present in those cases. Thus, there seems to be a multitude of mechanisms and different types of genetic regions involved in CPM, with genomic fragility as the unifying property. However, direct evidence of genomic fragility as a cause of CNV in CPM is still lacking and remains to be proven.

Although mosaicism for small CNVs confined to the placenta are extremely rare findings in CVS, misdiagnosis may occur unless a follow-up amniocentesis is performed.

## Data Availability

All data generated or analyzed during this study are included in this published article.

## Abbreviations

DMD: Duchenne muscular dystrophy
CVS: Chorionic villus sampling
SNV: Single nucleotide variant
CPM: Confined placental mosaicism
CNV: Copy number variation
GA: Gestational age
CMA: Chromosomal microarray
AC: Amniocentesis
DNA: Deoxyribonucleic acid
NIPT: Noninvasive prenatal testing
TFM: True fetal mosaicism
